# The HIV epidemic in Greenland – a slow spreading infection among adult heterosexual Greenlanders

**DOI:** 10.3402/ijch.v72i0.19558

**Published:** 2013-02-19

**Authors:** Karen Bjorn-Mortensen, Karin Ladefoged, Niels Obel, Marie Helleberg

**Affiliations:** 1 Department of Internal Medicine, Queen Ingrid's Hospital, Nuuk, Greenland; 2 Department of Infectious Diseases, University Hospital of Copenhagen, Rigshospitalet, Denmark

**Keywords:** Greenland, HIV, demographics, mortality rate, causes of death

## Abstract

**Introduction:**

We aimed to characterise the HIV epidemic in Greenland and to determine incidence, prevalence, mortality rates (MR) and specific causes of deaths.

**Study design:**

The study design used was population-based nationwide cohort study.

**Methods:**

We included all patients diagnosed with HIV in Greenland before 2011. Data were obtained from patient files, death certificates and the mandatory reports of HIV cases.

Incidence and prevalence were estimated as cases/100,000 adults/year and MR as deaths/1,000 person-years (PYR). MRs were estimated for the pre-HAART (≤1996), early-HAART (1997–2004) and late-HAART (≥2005) periods. Deaths were considered AIDS related, if CD4 count <6 months before death was <200 cells/µL and/or an AIDS-related event occurred <12 months of death.

**Results:**

We identified 171 cases of HIV among adult Greenlanders. Of these, 133 (78%) were infected in Greenland, 17 (10%) in Denmark and 21 (12%) in other places. The majority was infected through heterosexual contact [127 (74%)], 30 (18%) through homosexual contact, 3 (2%) through intravenous drug use and 11 (6%) through other or unknown routes of transmission.

The median age at HIV diagnosis was 46 years (interquartile range 34–56). The incidence increased from 3.8 before 1989 to 29.7 cases/100,000 adults/year in the late 1990s. The incidence has slowly declined to approximately eight cases/100,000 adults/year. Prevalence increased to a maximum in 2009 (174.9/100,000 inhabitants), and slowly declined since then. A total of 79 have died and 25 have emigrated. MRs were high in the pre- and early-HAART periods, 65.3 [95% confidence intervals (CI) 40.0–106.6] and 87.0 [95% CI 63.5–119.0], and a large fraction of deaths were AIDS related. In the late-HAART period, MR has declined markedly to 53.4 (95% CI 35.8–79.7) with a substantial decline in AIDS-related MR.

**Conclusion:**

Heterosexual contact is the main route of HIV infection and the patients are diagnosed at a median age of 46. The incidence of newly diagnosed HIV patients has decreased markedly since year 2000. Mortality is high although declining in recent years.

More than 25 years ago, the first case of HIV infection in Greenland was identified. Initially, the virus was introduced from Denmark, through homosexual and heterosexual contacts (1, 2). HIV infection spread fast primarily through heterosexual transmission amongst an older sub-population of individuals with low socio-economic status (3–6). A thorough description of the epidemic is provided in the supplementary material online.

A very high incidence of sexually transmitted diseases and induced abortions in Greenland (7) suggests that risk-taking behaviour is common among the general population, and it constantly raises concern of an increasing HIV incidence and a HIV epidemic spreading to the younger population.

The aim of the present study was to characterise and to describe the HIV epidemic in Greenland from the first case of known HIV infection until today and to determine if the HIV epidemic is spreading to other groups in the Greenlandic population. In addition, we determined mortality rates (MR) and causes of death among the Greenlandic HIV patients and how these changed over time with the introduction of HAART in the mi-1990s.

## Materials and methods

In a population-based cohort study, we estimated HIV incidence and MR of HIV-infected individuals in the period 1980–2011. Changing trends in mortality and causes of death were evaluated.

### Study population and period

We included all adult patients diagnosed with HIV in Greenland in the period 1980–2011. Two children, who were perinatally infected with HIV, were excluded to make the data comparable to other HIV cohort studies.

### Setting

Greenland has a population of approximately 56,000 people; of these, around 15,000 live in Nuuk. The rest of Greenland's population live in smaller towns or villages. Contact with the health care system is either through a local nurse or a medical clinic in the nearest town. All patient charts are stored electronically since 2007. Prior to this, files were kept on paper.

Results of laboratory analyses from all of Greenland have been stored electronically on a central server since 2001.

HIV patients in Nuuk are seen on an outpatient basis at the Department of Internal Medicine in Nuuk. This has been systemised since 2003. In some of the larger towns, a doctor travelling from Nuuk has carried out outpatient consultations every 6 or 12 months. Some patients from very remote districts travel to Nuuk for an outpatient consultation every 6 months, others have CD4 counts and HIV–RNA measured locally. Since 1996, HAART has been available free of charge at local health care centres or hospitals. When HIV patients are diagnosed, medications are delivered to the local health care centre for the treatment of the specific patient.

### Data sources

Data were collected by a systematic review of medical files, death certificates and files of the Greenland health authorities. As described in the supplementary material online, there is a mandatory notification of all HIV patients to the chief medical officer at the time of diagnosis.

The majority of Greenlandic HIV patients were already included in The Danish HIV Cohort Study, described in details elsewhere (8). Data include demographics, date of HIV infection, AIDS-defining events, antiretroviral treatment, CD4 cell counts and HIV–RNA measurements. By reviewing all reports ever made to the Greenlandic Health Officer, we identified 47 new cases not previously enrolled, since the Danish HIV Cohort Study only included patients diagnosed between 1995 and 2007.

Data on migration and vital status were obtained from the Central Civil Registration System, which includes all residents in Greenland and Denmark. Data on causes of death were obtained from death certificates stored at the office of the Greenlandic Health Officer. Data on Greenland's population were obtained from Greenland Statistics (9).

We used the unique 10-digit civil registration number assigned to all Danish and Greenlandic citizens to link data sources.

### Definitions

Areas of reported HIV cases were divided into Nuuk, Sisimiut and the rest of Greenland. Advanced HIV at time of diagnosis was defined as AIDS at time of diagnosis and/or a CD4 count <200 million cells/L within the first 6 month after diagnosis. Accordingly, late presenters were defined as patients with AIDS at time of diagnosis and/or a CD4 count <350 million cells/L within first 6 month after diagnosis. All deaths caused by accidents, injury or suicide were categorised as unnatural. The remaining deaths were either categorised as AIDS-related or non-AIDS-related deaths. Deaths were considered as AIDS-related deaths, if the last CD4 count measured <6 months before death was <200 cells/µL and/or an AIDS-related event occurred within 12 months of death. If the death certificate stated cause of death as AIDS and no other cause was found, the deaths were also categorised as AIDS related. All other deaths were categorised as non-AIDS related. Each of the three groups were divided into specific subgroups to examine the exact cause of death within each group.

All specific illnesses, which contributed to the chain of events leading to death, were included in analyses, that is, more than one cause could be included for the death of each patient.

### Statistic analysis

Time was calculated from the date of HIV diagnosis until death, emigration or 1 September 2011, whichever occurred first. Incidence and prevalence were calculated as number of cases and persons living with HIV/100,000 inhabitants >15 years of age/year. MRs were calculated as number of deaths/1,000 person-years (PYR) and 95% confidence intervals (CI) were estimated. The all-cause MR for the study period in total, as well as for three different periods stratified by year of diagnosis were estimated, the periods being the pre-HAART period (≤1996), the early-HAART period (1997–2004) and the late-HAART period (≥2005). When analysing CD4 counts at time of diagnosis and death, only those who had a CD4 count measured <6 months after diagnosis or <6 months before death were included in the analysis.

Kaplan–Meier methods were used to visualise mortality in Nuuk versus the rest of Greenland. Hazard ratios (HR) were estimated using Cox regression, adjusted for age at diagnosis, gender, mode of transmission and race. Also Kaplan–Meier methods were used to visualise mortality in the pre-HAART, early-HAART and late-HAART periods.

Statistical analyses were performed using SPSS statistical software, Version 15.0 (Norusis; SPSS Inc., Chicago, Illinois, USA) and Stata, Version 11 (Stata Corporation, College Station, Texas, USA).

Studies on the Danish HIV Cohort are approved by the Danish Data Protection agency, the approval also included registration of the Greenlandic HIV patients.

## Results

### Characteristics

Until 1 September 2011, a total of 171 adults (112 males and 59 women) have been reported infected with HIV in Greenland (Table I). In addition, 2 children were perinatally infected with HIV in 1989 and 1990, respectively. One of these children died of AIDS shortly after birth and the other moved to Denmark and is therefore lost to follow-up. Of the 171 adults, 25 have emigrated, 79 have died in Greenland and 67 are currently living with HIV in Greenland. Of the 25 emigrated persons, 11 emigrated in the pre-HAART, 8 in the early-HAART and 6 in the late-HAART period. Eighteen were male and 16 were below 35 years of age. Sixteen were infected by heterosexual contact, 9 by homosexual contact, 1 by intravenous drug abuse, 1 by blood transfusion and 3 by an unknown route of transmission. Eleven were from outside Nuuk or Sisimiut.

**Table I:** Characteristics of the HIV population in Greenland, N (%)

	Total
Total^a^	171
Died in Greenland	79 (46.2)
Living with HIV in Greenland	67 (39.2)
Emigrated	25 (14.6)
Gender	
Males	112 (65.5)
Females	59 (34.5)
Race	
Greenlandic	145 (84.8)
Caucasian	15 (8.8)
Unknown	8 (4.7)
Asian	2 (1.2)
Negroid	1 (0.6)
Mode of transmission	
Heterosexual	127 (74.3)
Homosexual/bisexual	30 (17.5)
Unknown	9 (5.3)
Injection drug abuse	3 (1.8)
Blood transfusion	2 (1.2)
Perinatal	+2
Place of transmission	
Greenland	133 (77.8)
Denmark	17 (9.9)
Unknown	8 (4.7)
Asia, Africa, Europe, Middle East	13 (7.6)
Town of residency	
– Town of transmission	
Nuuk	89 (52.0)
– Local transmission	71
Sisimiut	43 (25.1)
– Local transmission	40
Tasiilaq	8 (4.7)
– Local transmission	5
Rest of Greenland	31 (18.1)
– local transmission	17
Median age (years)	46 (IQR 34–56)
Advanced HIV^b^	31 (18.1)
Late presenters^c^	68 (39.8)

a Two children perinatally infected not included in the analysis.

b If AIDS at diagnosis and/or CD4 count <200 measured less than 6 months after diagnosis.

c If AIDS at diagnosis and/or CD4 count >350 measured less than 6 months after diagnosis.

**Fig. 1:**
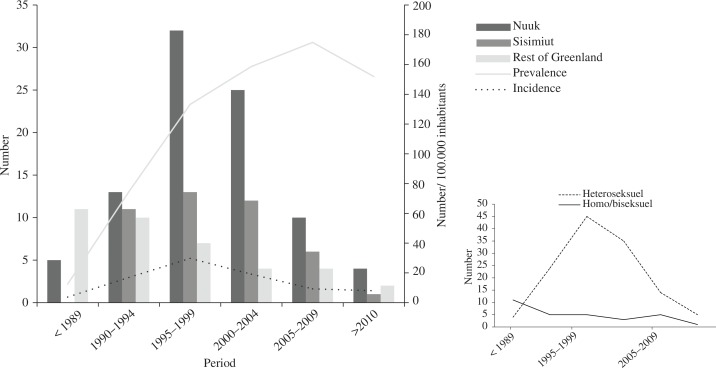
Area of reported HIV cases, incidence and prevalence/100,000 inhabitants >15 years of age. Number of persons reported with HIV in Nuuk, Sisimiut and the rest of Greenland.

Of the 171 cases identified, 145 (84.8%) were Greenlandic (Table I), 133 (77.8%) were infected in Greenland, 17 (9.9%) were infected in Denmark and 21 (12.3%) at other or unknown locations. The majority were infected through heterosexual contact [127 (74.3%)], while 30 (17.5%) were infected through homosexual contact, 3 (1.3%) through intravenous drug use and 11 (6.5%) had other or unknown routes of transmission. In the two largest towns in Greenland, Nuuk and Sisimiut, 89 (52.0%) and 43 (25.1%) persons, respectively, have been diagnosed with HIV. Transmission is mainly seen in these towns, whereas in the rest of Greenland transmission is rare (Fig. 1). Of the 38 persons diagnosed with HIV outside Nuuk and Sisimiut, 12 were diagnosed in a small epidemic on the East Coast before 1995. Since then, no transmission has been recognised on the East Coast. Excluding two children, the median age of HIV diagnosis was 46 years [interquartile range (IQR) 34–56, range 20–82]. In the period before 1989, the median age at diagnosis was lower [28 years (IQR 27–33)] and homosexual transmission higher (65%) than in the later periods. When looking through the early reports, we noticed a trend of married men transmitting HIV to both male contacts and their wives, especially on the isolated Eastern coast.

### Incidence and prevalence

The HIV incidence rose from 3.8/year/100,000 inhabitants before 1989 to 29.7/year/100,000 in the period 1995–1999. Since 2000, the incidence has decreased and is now 8/year/100,000 (Fig. 1). The prevalence increased significantly until the end of the 1990ties. Thereafter the prevalence still increased but at a somewhat slower rate. The highest prevalence was observed in the period 2005–2009 (174.9/100,000 inhabitants), after which it declined slightly. In 2010–2011, the prevalence was 151.9/100,000.

### Mortality

Data concerning mortality are indicated in Table II. Before 1 September 2011, 79 HIV patients died in Greenland [MR 69.1 deaths /1,000 PYR (95% CI 55.4–86.2)]. Of these, 43 (54.4%) died of AIDS-related causes, 32 (40.5%) of non-AIDS-related causes and 4 (5.1%) had unnatural deaths. The median age at time of death was 57.4 years (IQR 47.4–65.0), the median CD4 count at time of death was 190 (IQR 40–447) and 47 (62.0%) had started HAART. The specific causes of death are shown in Table II. Of the 79 patients dying in Greenland, 8 were infected through homosexual contact. The mortality was high in the pre-HAART and early-HAART periods [MR 65.3/1,000 PYR (95% CI 40.0–106.6) and 87.0/1,000 PYR (95% CI 63.5–119.0)], and decreased to 53.4/1,000 PYR (95% CI 35.8–79.7) in the late-HAART period, with an increasing median age at time of death (pre-HAART 53.4 (IQR 41.2–60.8), early-HAART 52.0 (IQR 46.2–61.4) and late-HAART 65.0 (IQR 59.1–70.2) years). The AIDS-related MR was 36.7/1,000 PYR (95% CI 19.1–70.6) in the pre-HAART period, 53.5.1/1,000 PYR (95% CI 35.9–79.8) in the early-HAART period but only 22.3/1,000 PYR (95% CI 12.0–41.4) in the late-HAART period. The MRs of non-AIDS-related deaths and unnatural deaths remained fairly steady throughout the study period [28.0/1,000 PYR (95% CI 19.8–39.6) and 3.5/1,000 PYR (95% CI 1.3–9.3)].

**Table II:** Number of deaths, mortality rates (MR) and causes of death for all HIV patients dying in Greenland in the study periods stratified by year of HIV diagnosis

	Complete period	Pre-HAART	Early-HAART	Late-HAART
Total number of deaths^a^ (n)	79	16	39	24
Total observational time (years)	1143.3	245.0	448.5	449.4
MR/1,000 PY (95% CI)	69.1 (55.4–86.2)	65.3 (40.0–106.6)	87.0 (63.5–119.0)	53.4 (35.8–79.7)
Median last CD4 count before death^b^ (IQR)	190 (65–366)	137 (40–447)	190 (60–420)	250 (97–505)
On HAART before death [n (%)]	47 (62.0)	0	26 (66.7)	21 (87.5)
Median age at time of death [years (IQR)]	57.4 (47.4–65.0)	53.4 (41.2–60.8)	52.0 (46.2–61.4)	65.0 (59.1–70.2)
AIDS-related deaths [n (%)]	43 (54.4)	9 (56.3)	24 (61.5)	10 (41.7)
MR/1,000 PYR (95% CI)	37.6 (27.9–50.7)	36.7 (19.1–70.6)	53.5.1 (35.9–79.8)	22.3 (12.0–41.4)
– Aids-defining illness <1 year before death	28			
– CD4cells <200 at time of death	19			
– Opportunistic infections^c^	12			
– HIV wasting	9			
– Kaposi sarcoma	3			
– Lymphoma	2			
Non-AIDS-related deaths [n (%)]	32 (40.5)	6 (37.5)	13 (33.3)	13 (54.2)
MR/1,000 PYR (95% CI)	28.0 (19.8–39.6)	24.5 (11.0–54.5)	29.0 (16.8–49.8)	28.9 (16.8–49.8)
– Cancer^d^	8			
– Infections^e^	8			
– Other^f^	8			
– Unknown	4			
– Cardiovascular^g^	4			
Unnatural deaths [n (%)]	4 (5.1)	1 (6.3)	2 (5.1)	1 (4.2)
MR/1,000 PYR (95% CI)	3.5 (1.3–9.3)	4.08 (0.57–28.98)	4.5 (1.1–17.83)	(0.3–15.8)
– Suicide	2			
– Aspiration/drowning	2			

Pre-HAART (≤1996), early-HAART (1997–2004), late-HAART (≥2005).

a Patients known to have died after moving to Denmark are excluded from this analysis.

b Only included, if measured <6 months before death.

c *Pneumocystis carini* infections: 5, tuberculosis: 1, unknown: 6.

d Lung cancer: 4, oesophageal cancers: 2, cancer in the oral cavity: 1, cerebral cancer: 1, stomach cancer: 1.

e Pneumonia: 4, urinary tract infection: 1, gastroenteritis: 1, sepsis: 1, unknown infection: 1.

f Alcohol related: 2, bowel perforation: 2, chronic obstructive lung disease: 1, cerebral haemorrhage: 1, other bleeding: 1, age: 1.

g Acute myocardial infarction: 3, cardiac arrest: 1.

The median CD4 count at time of death increased from 137 (IQR 40–44) in the pre-HAART period to 250 (IQR 97–505) in the late-HAART period. In the early-HAART period, 66.7% were on HAART when dying whereas 87.5% had started HAART before death in the late-HAART period.

**Fig. 2:**
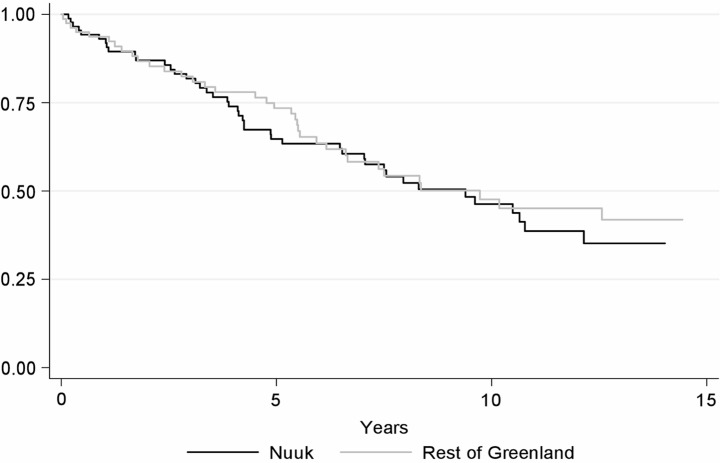
Kaplan–Meier curve showing survival of HIV patients living in Nuuk versus HIV patients living in the rest of Greenland in the total study period.

Figure 2 shows the survival of patients living in Nuuk versus the rest of Greenland HR 0.7 (95% CI 0.4–1.2).

## Discussion

We identified all persons diagnosed with HIV in Greenland since the first case was recognised in 1985. Age at diagnosis was in the interquartile range of 34–56 years and the route of transmission was primarily heterosexual. Most infected individuals were Greenlandic and most were infected in Greenland.

MR was high although declining in recent years and no significant differences were seen between mortality in Nuuk versus the rest of Greenland.

Early waves of transmission occurred either homosexually or bisexually but have changed into predominantly heterosexual transmission. This is possibly due to the small homosexual milieu in Greenland, making it attractive for homosexual individuals to move to a larger city, for example, Copenhagen. Also, no intravenous drug abuse exists in Greenland (10), resulting in zero transmission by this route. Only half of the persons infected are women, which could be explained by under-reporting of bisexual transmission or that some HIV-infected women transmitted the disease to more than one man. In the first clusters, many were infected bisexually, with men acquiring HIV through sex with men and transmitting it to their wives. Transmission happened mainly on the West Coast in Nuuk and Sisimiut, where the majority of the HIV-infected individuals are living today. Almost all patients from the East Greenlandic cluster have either died or moved to Denmark after receiving the diagnosis, and there have only been small clusters in other towns or settlements. Being infected by HIV in a small settlement could possibly result in social and sexual isolation, which is probably why transmission is no longer occurring there. Why transmission happened mostly among older adult individuals could be explained by simple coincidence: if HIV is introduced into this population and sexual contact is most common among age-matched individuals, transmission will most likely be limited to this population.

Despite a constant fear that the infection will spread to the population in general, the epidemic remains stable or slightly declining with a yearly incidence of approximately 8 new cases/year/100,000 inhabitants. Although a recent study estimates that approximately one-third of HIV-infected individuals on HAART have poor adherence (5), HAART may have played a role in containing the epidemic. The prevalence is slowly decreasing, which in the presence of declining MR reflects the declining incidence. The Greenlandic HIV incidence of 0.08% and prevalence of 0.15% is low compared to the prevalence of 0.4% in the European region and 0.8% worldwide, as estimated by WHO in 2010 (11), but approximately the same as the Danish HIV incidence of 0.05% and prevalence of 0.07% in 2005 (12).

The all-cause MR declined in the late-HAART period, most likely due to more effective treatment and therefore a declining rate of AIDS-related deaths. A larger fraction was on HAART (Table II) and the median CD4 count higher at time of death, all consistent with a more effective treatment of HIV patients. The rates of unnatural and non-AIDS-related deaths did not change throughout the study period. Surprisingly no significant difference in mortality was found between patients living in Nuuk versus the rest of Greenland, even though outpatient consultations are organised on a more regular basis in Nuuk. The numbers are small, and even though there was a tendency towards a lower mortality outside of Nuuk, the association was not statistically significant. This tendency could be attributed to a larger number of HIV-infected individuals living in smaller settlements moving to Denmark after diagnosis, which affects our estimates of MR.

In contrast to the low HIV incidence and decreasing prevalence are the incidences of other sexually transmitted diseases (STDs). The incidence of both chlamydia and gonorrhoea is extremely high in Greenland compared to Denmark (chlamydia >10 times higher, gonorrhoea >250 times higher in 2010), both incidences still rising (7). In 2009, more than 70% of chlamydia and gonorrhoea cases were among persons <25 years of age (7), suggesting a high level of sexual risk behaviour among young Greenlanders. Also, a high prevalence of *Mycoplasma genitalium* (13), hepatitis B (14, 15) and high abortion rates (7) adds to this perception.

In this scenario, it is quite interesting as to why HIV is still a low endemic disease in the Greenlandic population.

Maybe it is simply due to the fact that STDs in general are still most common amongst the young and HIV most common among a group of older adult individuals. But since most HIV transmissions occur heterosexually and a high number of STDs are also seen in the adult population, some transmission should be expected.

Many factors contribute to the rate of HIV transmission, for example, the prevalence, sexual habits, migration and travel, but also how contagious each patient is and for how long. If a patient is well treated on HAART, his/her viral load will be low, and it is highly unlikely that he/she will transmit the infection.

Our study confirms earlier findings, last evaluated in 2006, concerning age and mode of transmission (4–6). A recently published questionnaire survey among the Greenlandic HIV patients showed that 50% had only basic school education, 80% lived alone, 76% had a low income and 28% were addicted to hash (5), supporting earlier reports on HIV patients primarily belonging to an socially marginalised group (4). Also, 32% were self-reported sexually inactive (no sexual activity within the past year), while 8.6% reported unsafe sex within the past year, mostly from the non-adherent group (5). In our study, no parameters of social status were included, but the fact that HIV patients belong to this marginalised group probably explains some of the low transmission.

The large fraction of sexually inactive persons could explain some of the slow HIV transmission in Greenland, though an alarming number of non-adherent persons reported unsafe sex.

It has earlier been shown that HIV patients in Greenland are diagnosed at an early stage of infection (6), which is supported by our finding of only 18.1% having advanced HIV and/or AIDS at diagnosis and only 39.9% being late presenters. In a recent Danish study of the Danish HIV Cohort patients, the numbers were 34.7% and 51.2%, respectively (16).

Treatment of HIV patients in Greenland has been reported to begin at a later stage of disease and has been implemented at a slower pace compared to Denmark, yet showing marked improvements after 2003 (17). We found that among dying HIV patients, the CD4 count at time of death increased between the early-HAART period and the late-HAART period. In accordance, a larger proportion of patients were on HAART when dying, which also indicates improved management of HIV in Greenland.

High mortality among Greenlandic HIV patients before 2003 has been reported (17). Our study revealed that indeed mortality was high in the pre- and early-HAART periods but decreased dramatically since then. In a recent Danish study of mortality among HIV patients (18), all-cause MR declined from 135.5 to 16.4/1,000 PYR between 1995 and 2008. The relative all-cause mortality among Greenlandic patients was markedly lower in the pre-HAART period but higher in the late-HAART period. The reason for the relatively lower all-cause MR in Greenland in the pre-HAART period is possibly due to patients leaving Greenland before dying. Some of the higher MR in the late-HAART period of Greenlandic HIV patients compared to Danish HIV patients is explained by higher age of HIV patients in Greenland and the slightly lower life expectancy in Greenland in general. In 2010–2011, life expectancy in Denmark was 77.3 years for men and 81.6 years for women (19), while in Greenland it was 68.2 years for men and 72.9 for women (9) in 2007–2011.

The earlier mentioned questionnaire survey showed that only 67% of participants are adherent to treatment; of these more than 97% had viral load (VL) <200 copies/ml (5) The study reveals that even though treatment is sufficient among the treatment adherent patients, a large fraction of Greenlandic HIV patients are non-adherent and therefore at a greater risk of dying from their HIV infection.

The Greenlandic HIV Cohort is small with only 171 adults ever diagnosed with HIV and only 67 persons living with HIV in Greenland today, which limits the statistical significance of the analysis. We aimed to include all patients ever diagnosed with HIV in Greenland, and we are confident that we have fulfilled this purpose. We have included patients from the earlier clusters who were not included in previous reports (5, 6, 18) and all cases previously identified are included in the present study. A limitation in our analysis is the inconsistency with which HIV patients are seen as outpatients, especially outside of Nuuk (5). The small number of patients gives a statistical uncertainty, and since only patients attending outpatient consultations have CD4 counts measured, missing data for non-compliant patients is likely to bias analysis.

In conclusion, the HIV transmission in Greenland still occurs mostly heterosexually but is not, as previously described, limited to an elderly group of persons. Yet, the rate of transmission is low, and despite a possible high level of sexual risk-behaviour in the population, HIV incidence is declining slightly, and the epidemic is under control. Mortality among HIV patients in Greenland has declined substantially in recent years with a marked decline in AIDS-related deaths, probably due to improved treatment.
